# A Novel Bulk Planar Junctionless Field-Effect Transistor for High-Performance Biosensing

**DOI:** 10.3390/bios15030135

**Published:** 2025-02-22

**Authors:** Jeongmin Son, Chan Heo, Hyeongyu Kim, M. Meyyappan, Kihyun Kim

**Affiliations:** 1 Division of Electronics and Information Engineering, Jeonbuk National University, Jeonju 54896, Republic of Korea; thwjdals0820@naver.com (J.S.); ghlk4580@naver.com (C.H.); rhtmaehcl306@naver.com (H.K.); 2 Centre for Nanotechnology, Indian Institute of Technology Guwahati, Guwahati 781039, India; meyya@ieee.org; 3 Division of Electronic Engineering and Future Semiconductor Convergence Technology Research Center, Jeonbuk National University, Jeonju 54896, Republic of Korea

**Keywords:** BioFET, junctionless, two-terminal operation, biosensor, silicon on insulator, device simulation

## Abstract

Biologically sensitive field-effect transistors (BioFETs) have advanced the biosensing capabilities in various fields such as healthcare, security and environmental monitoring. Here, we propose a junctionless BioFET (JL-BioFET) for the high-sensitivity and low-cost detection of biomolecules and analyze it using detailed device simulations. In contrast to the conventional FET with junctions, the JL-BioFET simplifies fabrication by doping the source, channel and drain simultaneously with the same types of impurities, thereby reducing the fabrication effort and cost. Additionally, if the device is designed with optimal bias, it can operate with only the source and drain terminals, which reduces power consumption. Thus, cost reduction and reduced power consumption are strong motivations to pursue a new design. Therefore, we simulated two JL-BioFET structures (SOI JL, bulk JL) that operate without a gate electrode and compared their biosensing performances. The bulk JL-BioFET showed an average sensitivity three times higher than that of the SOI JL-BioFET across varying charge levels. Then, we optimized the sensing performance of the bulk JL-BioFET by adjusting three key parameters: the active layer thickness and the doping concentrations of the active layer and substrate. These encouraging results are expected to lead to future fabrication efforts to realize bulk JL-BioFETs for high-performance biosensing.

## 1. Introduction

Early diagnosis of diseases such as cancer increases survival rates by quickly detecting the relevant biomarkers in patients [1]. Early diagnostics is also an important part of public health strategies; for example, identifying infectious disease patients at the very early stage can prevent the widespread transmission of the infection. Therefore, early diagnosis methods, such as Polymerase Chain Reaction (PCR) and Enzyme-Linked Immunosorbent Assay (ELISA), have been extensively studied and utilized in practice [2,3]. However, their key disadvantages are the high cost of the measurement systems and long analysis times, which have prompted the investigation of various biosensor alternatives.

Recently, the biologically sensitive field-effect transistor (BioFET) has gained recognition as an alternative sensor because of its advantages such as its real-time response, the stability of the sensor characteristics and the ability to detect low concentrations of biomolecules [4,5]. Therefore, various materials such as silicon [6,7,8,9], carbon nanotubes (CNTs) [10,11], graphene [12] and other two-dimensional materials [13,14] have been utilized as active layers in BioFET construction to achieve enhanced sensitivity and low-power operation. Among the various candidate materials, silicon offers the advantages of proven large-scale production and low-power operation using CMOS fabrication.

The conventional silicon BioFET has a junction in the source-channel and drain-channel regions, requiring ion implantation and thermal annealing during device fabrication, making the process complex. A junctionless (JL) FET structure has long been advocated in the field of electronic devices and studied extensively to simplify device fabrication [15,16,17]. The introduction of this concept was prompted by the fact that the junctions get too close with the continued reduction in the feature size, resulting in steep doping gradients. The JL-FET, however, requires the use of a costly silicon-on-insulator (SOI) wafer.

**Figure 1:**
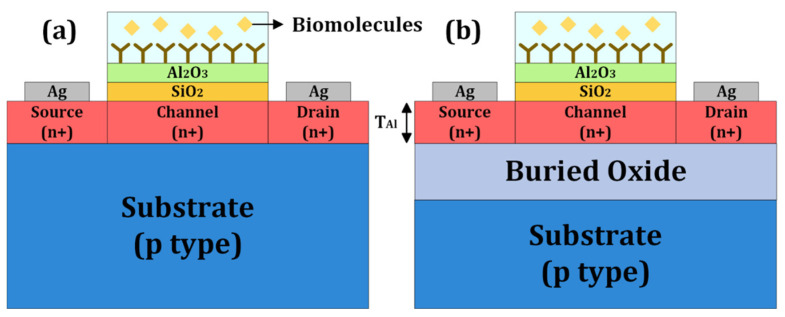
Schematic of the proposed (**a**) bulk and (**b**) SOI JL-BioFET structures.

Early adoption of JL-FETs for biosensing has been reported using silicon and CNT material systems [18,19,20]. Shukla et al. [18] fabricated planar silicon JL-FETs on an SOI wafer and demonstrated pH sensing. It would be desirable to eliminate the use of SOI, if possible. Here, we propose a bulk JL-BioFET that can be fabricated on a silicon wafer instead and validate its performance through Technology Computer-Aided Design (TCAD) simulation. Figure 1 shows a schematic of the bulk JL-BioFET used in the present simulation and an SOI JL-BioFET for comparison. The JL structure is created by doping the source, drain and channel with the same types of impurities. Additionally, only the source and drain terminals are utilized for operation by optimizing the voltage biasing, thereby reducing power consumption. The two-terminal operation implies that the reference electrode can be eliminated for the biosensing operation. The reference electrode, being bulky, hinders device miniaturization and is susceptible to damage during repeated biosensing operations, thereby compromising device reliability. All of these interesting features involving reduced processing steps can be expected to help with lowering the cost. In addition, the proposed device offers advantages in terms of miniaturization and improved reliability.

In this study, we simulated two JL-BioFET structures (SOI JL and bulk JL) without gate electrodes using the Sentaurus TCAD simulation tool and compared their sensing performances. The use of simulation tools is common in device design, regardless of the application, such as logic, memory, biosensors or gas sensors [4,17], as it is a cost-effective and time-saving approach prior to undertaking extensive fabrication endeavors. 

## 2. Two-Terminal JL-BioFET Operation

**Figure 2:**
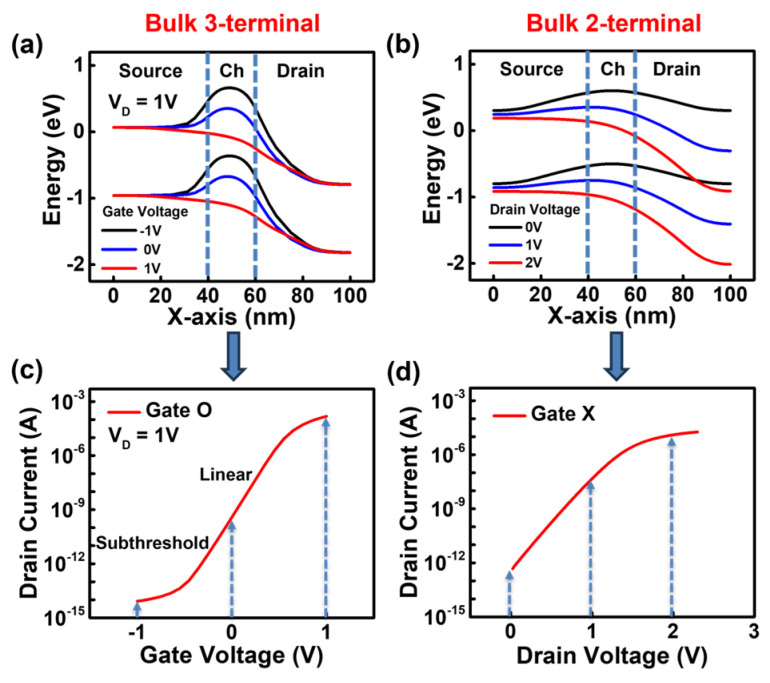
(**a**) Energy band diagram of 3-terminal bulk JL-FET. (**b**) Energy band diagram of 2-terminal bulk JL-BioFET at a charge of −1 × 10^12^ cm^−2^. (**c**) The *I_D_-V_G_* curve of the 3-terminal bulk JL-FET. (**d**) The *I_D_-V_D_* curve of the 2-terminal bulk JL-BioFET.

Figure 2a,c show the energy band diagrams and *I_D_-V_G_* characteristics of a three-terminal JL-FET with a gate. When a negative gate bias is applied in the case of a JL-FET with a gate, an electric field is created that pushes the electrons away at the SiO_2_/silicon interface. As a result, the conduction energy band of the channel rises, preventing electrons from easily moving through the channel, and the device turns off. When a positive bias is gradually applied to the gate, positive charges accumulate on the gate, pulling the electrons. Consequently, the conduction energy band lowers, allowing more electrons to flow through the channel, and the device switches to the on state.

Figure 2b,d show the energy band diagrams and *I_D_-V_D_* curve of the two-terminal JL-BioFET that operates only with source and drain and without a gate. When biomolecules are functionalized onto the channel above the gate dielectric, the negative charge of the biomolecules induces an electric field that pushes away electrons at the SiO_2_/channel interface. Similar to the three-terminal JL-FET with a gate, the elevated conduction band suppresses electron flow in the channel, allowing the JL-BioFET functionalized with biomolecules to function as a two-terminal biosensor. As the drain voltage increases when the energy barrier of the channel is raised due to the biomolecules, more electrons move from the source to the drain. However, based on the *I_D_-V_D_* curve, the drain current eventually saturates when the drain voltage becomes excessively high. To achieve high sensitivity in the BioFET, it is crucial to operate at an optimal drain voltage.

## 3. Device Design of JL-BioFET

### 3.1. SOI JL-BioFET vs. Bulk JL-BioFET

The SOI JL-FET is on an SOI wafer that consists of a thin active layer, a buried oxide (BOX) and a silicon handle wafer. This structure has the advantage of blocking leakage current to the substrate and reducing parasitic capacitance due to the BOX. Therefore, it is regarded as the most typical among the JL structures reported in various studies. However, SOI wafers are more expensive than standard Si wafers. Additionally, extremely thin nanowire channels (10–20 nm) and a high gate work function (5 eV) are required to turn the device off [16]. Thus, the SOI JL-FET has overall disadvantages.

In contrast, the bulk JL-FET has a structure where an n-type active layer is directly in contact with a p-type doped substrate. While the JL structure is in the lateral direction of the active layer, a junction is formed in the vertical direction between the active layer and the substrate. Therefore, the p-n junction in the vertical direction creates a depletion region and causes recombination, which substitutes for the role of the BOX in the SOI JL-FET. Additionally, the depletion region extending into the active layer reduces the effective active layer thickness compared to the physical active layer thickness, providing an advantage for the device’s off-state performance. Moreover, it is cost-effective because it does not require the use of expensive SOI wafers. Another advantage of the bulk JL-FET is the ability to control performance by adjusting the doping concentration and voltage biasing of the substrate [21].

### 3.2. Parameters Used in the Device Simulation

As shown in Table 1, all device parameters are identical except for the 90 nm BOX of the SOI substrate. The thickness of the active layer is set to a thin 20 nm in order to achieve the off state through carrier depletion in the active layer. There is no gate electrode, but a gate dielectric for biomolecule attachment is included. Al_2_O_3_ as a gate dielectric, with a deposition thickness of 2.5 nm here, allows for various chemical functionalizations and possesses high chemical stability. A 2.5 nm layer of SiO_2_ that adheres well to Al_2_O_3_ and minimizes the trap density is deposited in the layer below.

## 4. Simulation Method

### 4.1. Physics-Based Model

Various physics-based models were used in the simulation as described below. The Philips unified mobility model was utilized to account for mobility degradation due to impurity scattering and carrier–carrier scattering. The HighFieldSat and Enormal mobility models were also used to account for the degradation of carrier mobility due to high electric fields [22]. The oldSlotboom band gap narrowing model was utilized to account for the band gap narrowing effect caused by the high doping concentration in the active layer of the JL-FET [23]. SRH and Auger recombination were used to account for recombination between the active layer and the substrate [24,25,26]. The nonlocal BTBT (band-to-band tunneling) model was used to account for the BTBT between the valence band of the channel region and the conduction band of the drain region by constructing a predefined horizontal tunneling path [27].

**Figure 3:**
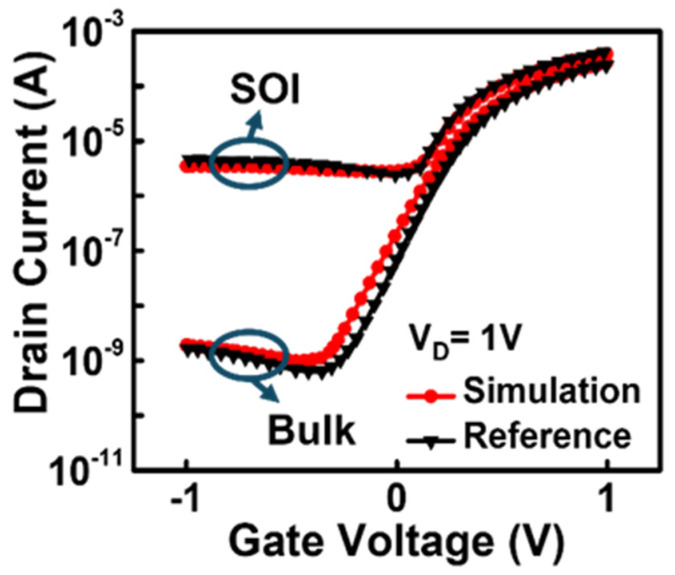
Model calibration through parameter adjustment of the BTBT model.

Figure 3 shows the results of the model calibration through parameter adjustment of the BTBT model. The calibration of the SOI JL- and bulk JL-BioFETs was first carried out against published simulation data of a bulk planar JL-FET with a gate electrode [28]. To match the reference results, the tunneling mass parameters of electrons and holes in the BTBT model were adjusted to 0.4 m_0_ and 0.65 m_0_, respectively. Moreover, the constant carrier lifetime utilized when there was no doping dependency (τ_max_) for electrons and holes was set to 1.6 × 10^−5^, and the reference doping concentration (*N_ref_*) was set to 1 × 10^17^ cm^−3^ to account for Shockley–Read–Hall recombination. Consequently, the carrier lifetime for electrons and holes corresponding to a doping concentration of 1 × 10^17^ cm^−3^ was set to 8 × 10^−6^ s [29].

### 4.2. Biosensing Model

The biosensing operation was modeled here by simply placing a specified charge on the gate dielectric surface. The concentration of the fixed charge representing the biomolecules in the simulation was set to −1 × 10^12^ cm^−2^, based on previous reports [30].

## 5. Results and Discussion

### 5.1. Electrical Characteristics

**Figure 4:**
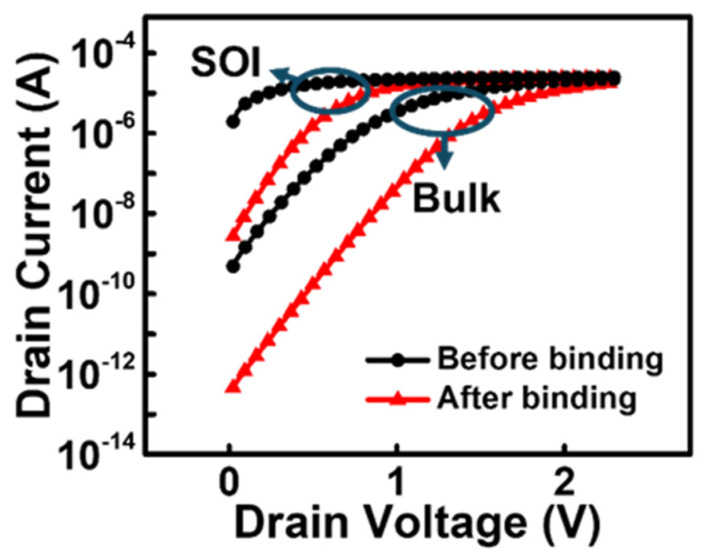
*I_D_-V_D_* characteristics of two types (SOI and bulk) of JL-BioFET.

Figure 4 shows the *I_D_-V_D_* curve of the proposed BioFET before and after the biosensing event. The drain voltage was swept from 0 to 2.5 V with no gate voltage applied and the source connected to the ground. The biosensing operation is well implemented in the simulation model, because current change is observed in both devices at the same drain voltage. When defining the switching ratio as the ratio of the current at a gate voltage of 0 V to the maximum current prior to biomolecule binding, the bulk JL-BioFET achieves a high switching ratio of ~10^6^, compared to ~10^4^ for the SOI JL-BioFET. This indicates that, within the typical operating voltage range (positive voltage range), the biomolecule attachment induces a larger current change, resulting in a broader dynamic range for biosensing applications.

**Figure 5:**
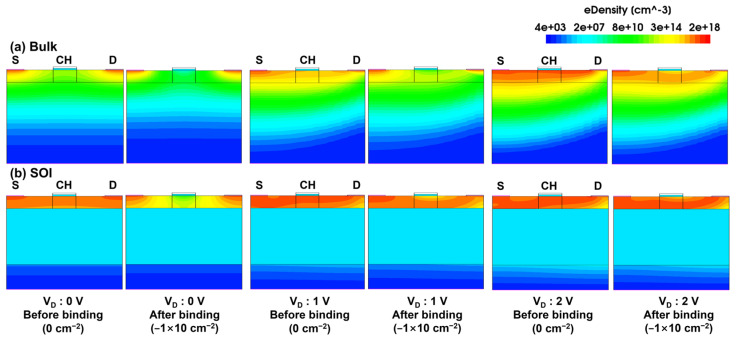
Electron density of (**a**) bulk and (**b**) SOI JL-BioFETs. In both cases, density profiles before and after binding for drain voltages of 0, 1 and 2 V are shown.

Figure 5 shows the electron density profile of the JL-FET with and without a negative charge (−1 × 10^12^ cm^−2^) for drain voltages ranging from 0 to 2.5 V. In both structures, when a negative charge is generated by the biomolecule at the Al_2_O_3_ surface at 0 V, it pushes electrons away in the channel. Therefore, the electron flow in the channel is blocked, resulting in a current reduction. Applying a positive voltage to the drain increases the electron density in the channel, lowering the energy band across the channel. This reduction in the energy barrier facilitates electron flow from the source to the drain, thereby turning the device on. The bulk device demonstrates superior turn-off control performance compared to the SOI device, as it achieves a deep depletion state in the channel at 0 V. This indicates that the bulk device can operate in a state closer to the “off state” even without applying a gate voltage.

### 5.2. Biosensing Performance

To evaluate the sensing performance of the designed device, sensitivity is defined as follows:

(1) $$Sensitivity=\left(I_{Do}-I_{D1}\right)/I_{D1}$$

**Figure 6:**
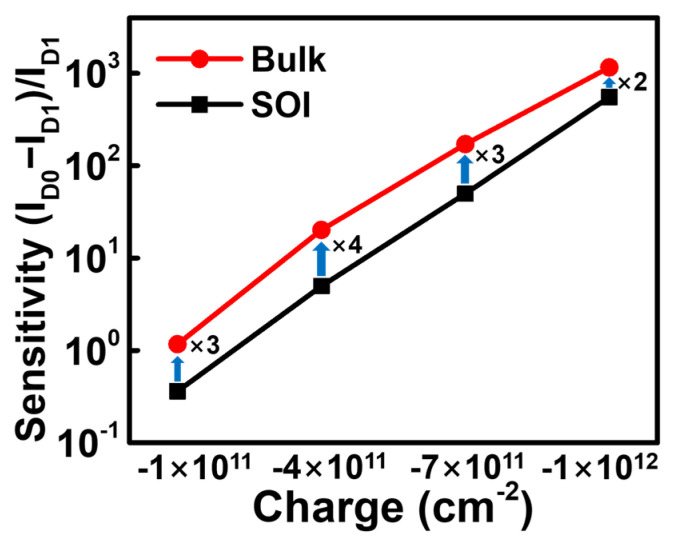
Sensitivity comparison between bulk and SOI JL-BioFETs with different surface charges.

Sensitivity is typically defined as the normalized current change relative to the current after the binding of biomolecules. *I_d_*_0_ is the baseline current, which corresponds to the *I_d_* of the functionalized device prior to the attachment of biomolecules. *I_D_*_1_ is the *I_D_* of the functionalized device after the biomolecule binding event [31]. The current change at a drain voltage of 0.1 V was utilized as a refence to extract the sensitivity. Figure 6 shows a sensitivity comparison of the two JL-BioFETs at different charges. The sensitivity values for the SOI JL-BioFET are 0.3, 4.9, 49.9 and 553 for charges of −1 × 10^11^ cm^−2^, −4 × 10^11^ cm^−2^, −7 × 10^11^ cm^−2^ and −1 × 10^12^ cm^−2^, respectively. The corresponding values for the bulk JL-BioFET are 1.1, 20.2, 171 and 1160, respectively. The sensitivity of the bulk JL-BioFET is higher than that of the SOI JL-BioFET for all surface charge densities. The SOI JL-BioFET has a current change of 5.59 μA at a drain voltage of 0.1 V during biosensing events, whereas the bulk JL-BioFET has a smaller current change of 1.54 nA. Although the current change of the SOI JL-BioFET due to the biosensing event is greater than in the bulk device, the sensitivity of the SOI JL-BioFET is lower than that of the bulk device because of its higher *I_D_*_1_. The observed performance difference can be explained as follows.

In the case of the SOI structure, the current flow of the channel is influenced only by the charge of the biomolecules bound to the surface. In contrast, the current flow of the channel in the bulk structure is affected not only by the charge of the biomolecules but also by the doping region formed beneath the channel. The doping region beneath the channel is formed with a type opposite to that of the channel, creating a depletion region, which effectively reduces the actual channel thickness. In this state, the charge induced by the biomolecules on the surface causes a greater change in channel resistance. Consequently, the doping region beneath the channel effectively reduces the actual channel thickness in the bulk structure, thereby enhancing sensitivity.

**Figure 7:**
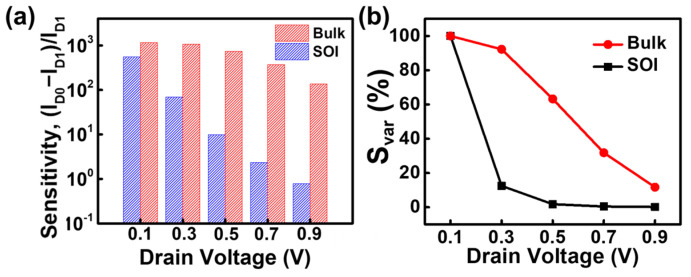
(**a**) Sensitivity as a function of the drain voltage for the proposed JL-BioFET at a surface charge of −1 × 10^12^ cm^−2^, and (**b**) sensitivity variation (*S_var_*) as a function of the drain voltage under a surface charge of −1 × 10^12^ cm^−2^.

Figure 7a shows the sensitivity as a function of increasing drain voltage at a surface charge of −1 × 10^12^ cm^−2^. The bulk structure consistently demonstrates higher performance at all drain voltages. The sensitivity decreases with increasing drain voltage in both structures. However, the sensitivity variation with respect to changes in the drain voltage is less sensitive in the bulk device compared to the SOI device. Figure 7b represents the sensitivity variation (*S_var_*) with respect to the drain voltage at a surface charge of −1 × 10^12^ cm^−2^. *S_var_* is defined as a percentage of the sensitivity at each drain voltage normalized to the sensitivity at a drain voltage of 0.1 V under a surface charge of −1 × 10^12^ cm^−2^. The *S_max_* for the bulk structure is greater than that for the SOI structure across all drain voltages. Notably, the sensitivity of the SOI device is more significantly influenced by changes in the drain voltage compared to the bulk device. When the drain voltage increases from 0.1 V to 0.3 V, the sensitivity of the SOI device drops to approximately 11%, and the sensitivity variation becomes negligible beyond 0.3 V. In contrast, the bulk device shows a linear decrease in sensitivity with increasing drain voltage. These results indicate that the biosensing performance of the SOI device significantly degrades by fluctuations in the drain voltage, leading to reduced reliability in biosensing applications. 

### 5.3. Optimization of Biosensing Performance in JL-BioFET

**Figure 8:**
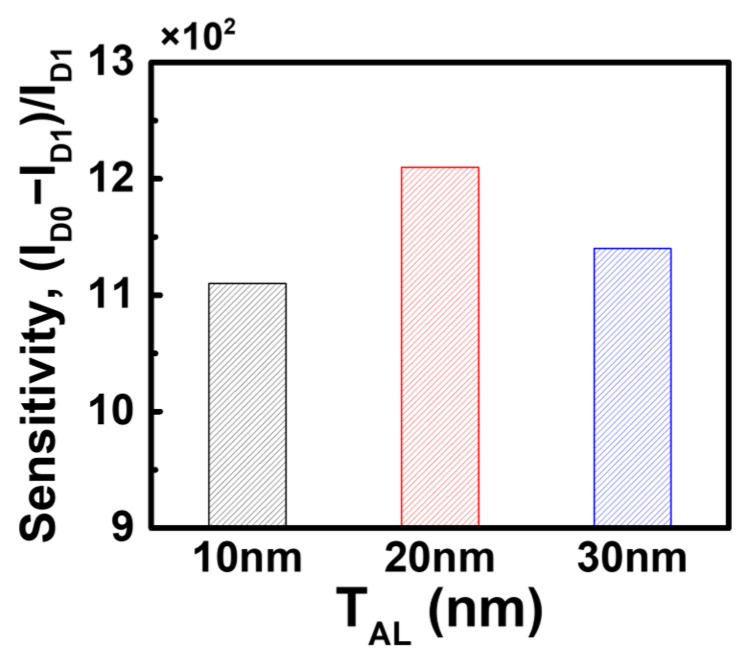
Sensitivity of the bulk JL-BioFET as a function of the active layer thickness.

The results above confirm that the bulk JL-BioFET demonstrates superior biosensing performance compared to the SOI structure in a two-terminal sensing operation. Next, we optimized the biosensing performance of the bulk JL-BioFET by varying the device parameters, including the thickness of the active layer (*T_AL_*) and the doping concentrations of both the active layer (*C_AL_*) and the substrate (*C_Sub_*). Figure 8 shows the sensitivity of the bulk JL-BioFET as a function of *T_AL_*. The highest sensitivity was observed at a *T_AL_* of 20 nm, exhibiting up to a 9% improvement compared to other active layer thickness values. When the active layer is too thin or too thick, the change in channel conductivity caused by biomolecule binding becomes less significant. The relatively lower sensitivity compared to a *T_AL_* of 20 nm is attributed to the relatively higher *I_D_*_1_. 

**Figure 9:**
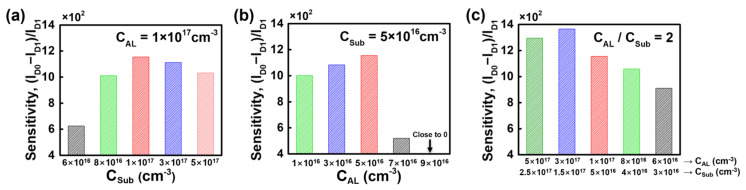
Sensitivity of the bulk JL-BioFET as a function of the (**a**) doping concentration of the substrate, (**b**) doping concentration of the active layer and (**c**) doping concentration of both the substrate and active layer, achieving C_AL_/C_Sub_ = 2.

Figure 9 shows the sensitivity variation of the bulk JL-BioFET with a *T_AL_* of 20 nm as a function of the doping concentration. Figure 9a illustrates the sensitivity as a function of *C_Sub_* with *C_AL_* fixed at 1 × 10^17^ cm^−3^. Figure 9b shows the sensitivity as a function of *C_AL_* with *C_Sub_* fixed at 5 × 10^16^ cm^−3^. Figure 9a shows the sensitivity peaks at 5 × 10^16^ cm^−3^ for the substrate doping concentration. When the doping concentration of the substrate increases over 5 × 10^16^ cm^−3^, the sensitivity variation is approximately 20%. However, when the concentration decreases below 5 × 10^16^ cm^−3^, the sensitivity variation becomes more significant. In particular, the sensitivity drops sharply by around 50% when the channel doping concentration decreases. When the substrate doping concentration is much lower than the channel doping concentration, the depletion region at the p-n junction formed between the substrate and the channel becomes thicker in the substrate than the channel, which degrades the channel modulation ability of the bulk device. 

The change in the channel doping concentration has a greater impact on the sensitivity than the doping concentration of the substrate. In particular, when the channel doping concentration is 3 × 10^17^ cm^−3^, the sensitivity drops sharply by about 57% and approaches zero at 5 × 10^17^ cm^−3^. This is because a higher channel doping concentration significantly reduces the influence of charge from the biomolecule binding. Therefore, the relationship between the doping concentrations of the channel and the substrate has a significant impact on the sensitivity. The sensitivity in both cases peaked at 1160 with a doping concentration ratio of 2 between the active layer and the substrate. Finally, the sensitivity was observed for various substrate and channel doping concentrations, with the doping concentration ratio fixed at 2 (Figure 9c). The highest sensitivity was obtained with the active layer doped at 3 × 10^17^ cm^−3^ and the substrate doped at 1.5 × 10^17^ cm^−3^.

The observed changes in the optimization exercise above can be understood by recognizing that the sensitivity in the bulk structure is affected by the actual channel thickness, as mentioned earlier. The thickness itself changes due to the doping region formed beneath the channel. Therefore, directly reducing the channel thickness and varying the doping concentrations of the channel and beneath the channel are the options for optimization.

## 6. Conclusions

We theoretically investigated two junctionless BioFET structures, namely, SOI JL and bulk JL, which offer advantages in terms of fabrication complexity. They both operate with only two terminals, source and drain, without a gate electrode, which helps to reduce power consumption and eliminate the reference electrode. The bulk JL structure generates an additional depletion region due to the vertical p-n junction between the active layer and the substrate, resulting in an average sensitivity performance that is three times higher compared to the SOI JL structure across varying charge levels. Therefore, we optimized the performance of the bulk JL-BioFET by varying three key parameters: the thickness of the active layer and the doping concentrations of both the active layer and the substrate. The highest sensitivity performance was achieved with an active layer thickness of 20 nm, active layer doping of 3 × 10^17^ cm^−3^ and substrate doping of 1.5 × 10^17^ cm^−3^. The impressive results here suggest the proposed bulk JL-BioFET as a suitable candidate for biosensing applications requiring high sensitivity and a worthy candidate for fabrication.

## Figures and Tables

**Table 1:** Parameters used in the device simulation.

Description (Parameter)	Values
Active layer thickness (*T_AL_*)	20 nm
SiO_2_ thickness (*T*_*SiO*_2__)	2.5 nm
Al_2_O_3_ thickness (*T*_*Al*_2_*O*_3__)	2.5 nm
Buried oxide thickness (*T_Box_*) [SOI]	90 nm
Source region length (*L_Source_*)	40 nm
Drain region length (*L_Drain_*)	40 nm
Gate dielectric length (*L_G_*)	20 nm
Active layer doping concentration (*C_AL_*)	1 × 10^17^ cm^−3^
Substrate doping concentration (*C_Sub_*)	5 × 10^16^ cm^−3^

## Data Availability

The data are contained within the manuscript.
